# Testing the Utility of an Integrated Analysis of Copy Number and Transcriptomics Datasets for Inferring Gene Regulatory Relationships

**DOI:** 10.1371/journal.pone.0063780

**Published:** 2013-05-30

**Authors:** Xin Yi Goh, Richard Newton, Lorenz Wernisch, Rebecca Fitzgerald

**Affiliations:** 1 Medical Research Council Cancer Cell Unit, Hutchison-MRC Research Centre, Cambridge, United Kingdom; 2 Department of Oncology, University of Cambridge, Cambridge, United Kingdom; 3 Medical Research Council Biostatistics Unit, Cambridge, United Kingdom; The Institute of Cancer Research, London, United Kingdom

## Abstract

Correlation patterns between matched copy number variation and gene expression data in cancer samples enable the inference of causal gene regulatory relationships by exploiting the natural randomization of such systems. The aim of this study was to test and verify experimentally the accuracy of a causal inference approach based on genomic randomization using esophageal cancer samples. Two candidates with strong regulatory effects emerging from our analysis are components of growth factor receptors, and implicated in cancer development, namely ERBB2 and FGFR2. We tested experimentally two ERBB2 and three FGFR2 regulated interactions predicted by the statistical analysis, all of which were confirmed. We also applied the method in a meta-analysis of 10 cancer datasets and tested 15 of the predicted regulatory interactions experimentally. Three additional predicted ERBB2 regulated interactions were confirmed, as well as interactions regulated by ARPC1A and FANCG. Overall, two thirds of experimentally tested predictions were confirmed.

## Introduction

To establish causal gene regulatory relationships, experimental manipulation of genes is usually required. Observational data on its own is, except in a few very special situations, insufficient. The main problem is that merely observed correlation between the expression of a gene A and of a gene B is causally confounded: it can be explained by a causal path from A to B, as well as one from B to A, or by a third gene C influencing both, or by external factors arising from experimental procedure or data preprocessing. To establish unambiguously that a causal path leads from gene A to gene B one has to manipulate or randomize gene A in order to break any causal path leading into A (either from B or C or external factors).

In certain circumstances, however, nature provides ‘natural’ randomization experiments. Studies utilizing ‘Mendelian randomization’ [1] in the search for causal genes in various genetic diseases [2], [3] are an example. Cancer genomes provide another example of natural randomization that we believe can be utilized in the inference of causal gene relationships. The heavily altered copy numbers of genes in the genomes of cancer cells provide natural gene dosage randomization.

In order to test the hypothesis that natural copy number variation, or ‘genomic randomization’, helps in inferring functionally significant regulatory interactions within cancer genomes we designed an algorithm that analyzes matched array comparative genomic hybridization (aCGH) and microarray gene expression profiling data.

Currently existing algorithms for integration of genome-wide data from matched genomic and transcriptomic data, such as ACE-it [4], GEDI [5], SLAMS [6] and VAMP [7] generally set out to identify correlations between copy number changes and differential gene expression levels at the same chromosomal loci, with the aim of investigating the *cis*-acting effects of gene dosage on gene expression alterations.

However Yuan et al. [8] integrate matched copy number and transcriptomic data from breast cancer samples to investigate regulatory relationships between gene pairs positioned at different loci in the genome, so-called *trans*-acting effects. Their work differs from the current study in the method of integration used and the indirect confirmation of their predictions through gene set enrichment studies rather than direct experimental validation of predicted regulations. Lee et al. [9] have also looked for *trans*-acting effects using the correlation of matched copy number and transcriptomic data, presenting evidence of predictions through gene set enrichment studies. Li et al. [10] have recently published related work. They use matched gene expression and copy number data to predict gene regulatory relationships, followed by siRNA experimental validation. Their work differs from the current study in the method of integration used, and in their applications. They use NCI-60 cancer cell lines, whilst we use primary human tumour samples for predicting regulatory relationships. Moreover, the experimental work concentrates on one predicted regulating gene, whilst the experimental work described here validates the predicted relationships of a number of regulating genes.

Other studies have used correlation between copy number variation and gene expression to find possible driver genes from matched aCGH and microarray gene expression data in cancer samples, that is, genes responsible for the breakdown of growth and proliferation control in cancer cells. Some studies add a second separate step, deriving gene regulatory networks from expression data involving driver genes (for example [11], and references therein). However, using expression profiles alone to derive causal regulatory links, even after identifying possible regulating genes using both aCGH and expression, is still prone to the problem of causal confounding. Even if there is some correlation between aCGH and expression profiles for gene A, much of the correlation between expression profiles of gene A and gene B could still be due to confounding.

Here we propose to tackle this problem directly by testing for the correlation not only of copy number of gene A with its own expression but also of copy number of gene A with the expression of gene B, excluding any possible confounding on the level of expression profiles only. We define a ‘regulating gene’ as one whose enforced, manipulated up or down expression has a direct or indirect effect on the up or down regulation of a ‘target gene’. Primary candidates for regulating genes which can be identified by integrating aCGH with expression data are genes having corresponding changes in their mRNA expression levels following copy number alterations. Candidate target genes are identified through the procedure described in the following paragraph. The key feature of a regulating-target gene pair is that expression levels of the target gene can be influenced by manipulating the expression of the regulating gene while keeping everything else as equal as possible. The regulatory relationship between regulating gene and target gene can be a direct relationship (of a transcription factor on its target gene) or a very indirect one through intermediate regulatory steps. In fact, one regulating gene of interest in this study is ERBB2, a component of the epidermal growth factor receptor, not a transcription factor. One of our aims is to provide a tool for improved understanding of the specific downstream transcriptional effects of genes at the top of signal transduction chains.

**Figure 1:**
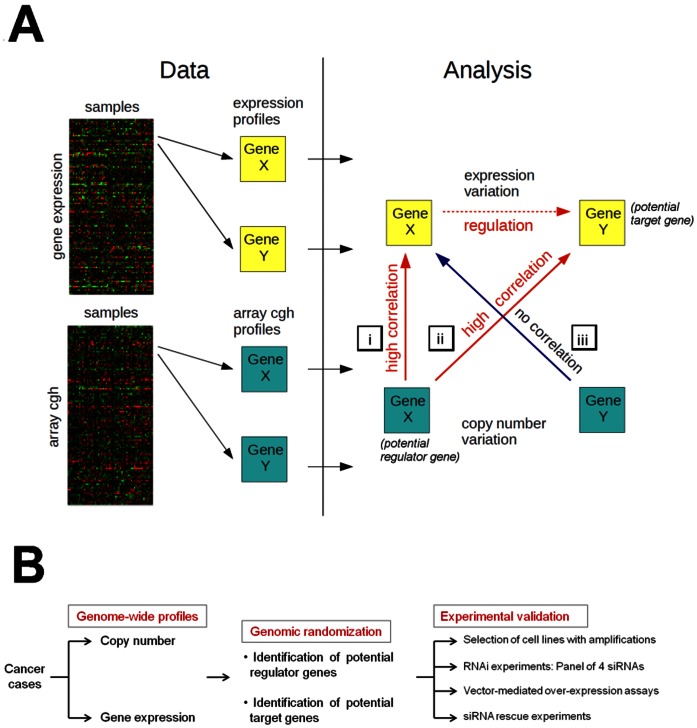
Schematic illustration of analysis. **A.** Starting with genome-wide data from array comparative genomic hybridization and microarray gene expression, potential gene regulations were identified based on three conditions, marked i–iii in the figure and described in detail in the text. **B.** Flow chart of steps involved to validate the predictions.

The genomic randomization algorithm is based on three conditions to identify potential interactions between regulating-target genes: i) expression changes of a potential regulating gene must correlate highly with its own aCGH status changes; ii) expression changes of a potential target gene must correlate highly with its regulating gene’s aCGH status changes; iii) the correlation between a regulating gene’s expression changes and its potential target gene’s aCGH status changes must be low. The last step is required since copy number variation not only affects the coding sequence for one gene but possibly many genes in the neighborhood on a genome level. Criterion iii excludes the possibility that the target gene is within such a neighborhood. We then used the outcome from statistical tests of these three correlations to rank the probability of a regulatory relationship for all gene pairs. Figure 1 illustrates the genomic randomization approach.

The problem of a third gene confounding the relationship between regulating gene and target is circumvented by using partial correlation conditioning on all other genes except the regulating gene and target. We performed the analysis twice, using partial correlation and using Pearson correlation. We compared the results of the two analyses with the results of our experimental tests of the predicted interactions in order to assess the importance of using the computationally more complex partial correlation analysis.

The association between copy number and gene expression will in general be complex, the exact relationship being gene specific. In this study we were interested in investigating by experimentation potential regulator-target pairs that were identified as having a linear association between copy number and gene expression.

To test experimentally the ability of genomic randomization in predicting potentially functional gene interactions, we adopted a strategy that involved RNA interference (RNAi) experiments targeted by a panel of four small interfering RNAs (siRNAs). All experiments were performed in carefully selected cell lines harboring amplifications of potential regulating genes to investigate the effects of silencing selected regulating genes. Having experimentally validated all five gene relationships predicted by genomic randomization based on an initial dataset (54 samples), we further tested and experimentally validated our method using genome-wide data from a compilation of ten publicly available cancer datasets (580 samples in total) consisting of different tissue types with matched copy number and gene expression data.

We propose our approach as an instrument to dissect the effects of novel regulating genes with important downstream target genes; in particular the effects of receptors, as shown here for ERBB2 and FGFR2, two examples of growth factor receptors. The downstream regulatory effects of growth factor receptors are only understood in very broad terms. The main problem is that many receptors affect similar signal transduction pathways, such as ERK, MAPK, or Akt signalling. However, different receptors can have markedly different effects on the gene expression profile of cells, which is only partially reflected in the current knowledge of signalling pathways.

All data utilized and analyzed in this study are publicly available in online databases (sources cited in Table S1 in File S2). The code in R can be found in file Code S1.

The aim of this study was to test and verify experimentally the accuracy of a causal inference approach based on matched copy number and transcriptomics datasets from cancer samples. Our results clearly demonstrate the value of a detailed statistical analysis of high-throughput data on cancer samples to unearth crucial information about the missing details of biological processes such as regulation through signal transduction in cancer. The percentage of true positives is high enough for the results to be useful as suggestions for experimental investigation or to be included with other evidence in probabilistic models of gene regulatory networks.

## Materials and Methods

### Data

Data analyzed included two previously reported studies of our own [12], [13], in addition to eight others which were obtained from online public databases (Table S1 in File S2). Data from one of the 10 studies was generated on two different expression platforms, and data from each of these platforms was treated separately, so altogether 11 sets of data from a total of 10 studies were included in the multiple dataset analysis.

### Statistical Analysis

Matched array comparative genomic hybridization and microarray gene expression profiling data were integrated and analyzed using the R statistical environment [14] and the R package ‘GeneNet’ (http://cran.r-project.org/web/packages/GeneNet) [15]. Genes in a dataset that are worth investigating as potential regulating genes must have a high correlation between their own aCGH status and own expression, indicating that copy number changes are influencing the gene’s expression. For each of the potential regulating genes we look for potential target genes, which have a high correlation between the target gene’s expression and the regulating gene’s aCGH status. An alternative approach would be to look for target genes which have a high correlation between the target gene’s expression and the regulating gene’s expression. Correlating expression profiles from within the same experiment can however result in false correlations; arising from the experimental design or execution, or from data pre-processing such as normalization.

The aCGH data was not thresholded for the analysis so that variations in aCGH values less than a single amplification were taken into account. Such variations are relevant because of the heterogeneity of the cancer samples being studied. By using matched aCGH and expression profiles we eliminated the effects of a sample’s heterogeneity considering that both sets of data were affected equally.

We set three conditions to identify potential regulatory relationships between regulating genes and target genes: i) Expression changes of a potential regulating gene must correlate highly to its own aCGH status; ii) Expression changes of a potential target gene must correlate highly to its regulating gene’s aCGH status; iii) The correlation between a regulating gene’s expression changes and its potential target gene’s aCGH status must be low. In practice we used the general rule that the *p*-value for condition iii must simply be greater than that for condition i in order not to exclude regulator-target pairs with some degree of co-amplification from the lists of predicted interactions. These three conditions gave three corresponding significance levels for each gene pair, which were used to rank gene pairs according to the probability of a potential regulatory relationship.

For each dataset the aCGH data was location and scale normalized using the median and mad, and the expression data either median and mad normalized if the data distribution was approximately normal, or Gaussian normalized if not. The aCGH and expression probes were mapped by probe names to give the maximum number of genes with corresponding aCGH and expression profiles (Table S1 in File S2).

For all experiments performed in this study all cited *p*-values are adjusted by the false discovery rate (fdr) method of Benjamini-Hochberg (R function p.adjust with method parameter ‘BH’).

#### Single dataset: Genomic randomization analysis

For the analysis of the single, esophageal adenocarcinoma (EAC), dataset, for each gene pair, three partial correlations were calculated corresponding to each of the three conditions. The partial correlations were calculated using the R package ‘GeneNet’ and each correlation’s *p*-value found using a randomization of the dataset as a null distribution. The randomization of the dataset was achieved by randomizing the probe labels of the matrix of expression values and is described further in the section ‘Randomization’ below. The *p*-values were adjusted for multiple testing using the fdr method of Benjamini-Hochberg (R function p.adjust with method parameter BH). For computational reasons only a subset of probes were included in the initial analysis; 2,000 probes with the highest variance of their expression profiles were selected.

#### Multiple datasets: Genomic randomization analysis

For the analysis of the multiple datasets, in order to include more than 2,000 probes in the analysis, initially Pearson correlations rather than partial correlations were calculated, using the same three conditions described above. Those genes with probes present in all 11 data sets were identified, giving a list of 1,410 probes. The *p*-values of the Pearson correlations between each probe’s aCGH profile and its own expression profile were calculated for the 1,410 probes in each of the 11 datasets using Fisher’s Z transform (R function cor.test). The alternative hypothesis that the correlation is greater than zero was tested. The 11 correlation *p*-values for a probe were combined using Fisher’s method for combining *p*-values (R function survcomp::combine.test). The significance of the combined *p*-values was calculated using a randomization of the datasets to generate a null distribution of combined *p*-values (see below). The resulting permutation combined *p*-values were adjusted for multiple testing using the false discovery rate method of Benjamini-Hochberg, to give an fdr for each probe based on its aCGH-expression correlations in all 11 datasets. In addition, which of the 11 datasets indicated an aCGH-expression correlation was assessed for each of the 1,410 probes using an arbitrary threshold of 0.05 on a probe’s 11 correlation *p*-values.

The 1,410 probes were ranked according to the permutation combined *p*-value of the aCGH-expression correlation and the number of datasets (N) in which the gene showed an aCGH-expression correlation. The highest ranked genes for which there are known cell lines containing amplifications of these genes (CONAN - Cancer Genome Project, Wellcome Trust Sanger Institute: http://www.sanger.ac.uk/cgi-bin/genetics/CGP/conan/search.cgi) were selected for further investigation as potential regulating genes; namely ERBB2, FANCG and ARPC1A. We selected genes for which there are known cell lines containing amplifications of these genes to obviate the necessity of artificially inducing an amplification and over-expression phenotype, hence reducing the potential for in vitro bias.

Each of the three genes selected as potential regulating genes were analyzed independently for potential target genes using conditions ii and iii above. These two steps of the analysis were applied to all potential target genes in each dataset (i.e all other genes in the dataset, not just the initial 1,410 genes that had probes in all 11 datasets). The *p*-values of the correlation of the potential regulating gene’s aCGH status with the expression profiles of all potential target genes were calculated (condition ii). We tested separately the two alternative hypotheses; that the correlation is greater than zero and that the correlation is less than zero. This was done for each of the N datasets in which the potential regulating gene showed an aCGH-expression correlation. The *p*-values of the correlation of all potential target genes’ aCGH status with the potential regulating genes’s expression were also calculated (condition iii), again for each of the N datasets in which the potential regulating gene showed an aCGH-expression correlation.

Next, we combined the correlation *p*-values using Fisher’s method, assessed their significance level using a null distribution of combined *p*-values derived from a randomization of the datasets (see below) and the resulting permutation combined *p*-values fdr adjusted using the method of Benjamini-Hochberg. The number of datasets in which the gene pair may have a relationship was assessed by using an arbitrary threshold of 0.05 on the correlation *p*-values. This number was relevant in order to ensure no single dataset or cancer type was introducing bias to the analysis.

For each of the three potential regulating genes the potential target genes were filtered by the permutation combined *p*-value of the correlation between the regulating gene’s expression and the target gene’s aCGH status, which had to be low. The genes were then ranked according to the permutation combined *p*-value of the correlation between the regulating gene’s aCGH status and the target gene’s expression changes, and by the number of datasets in which the correlation *p*-values indicated that the gene pair had a relationship. The highest ranked gene pairs were selected for experimental validation (provided that primers could be optimised for the target genes).

Subsequently, post-experimental validation, we repeated the analysis but using partial instead of Pearson correlations. For computational reasons this analysis was restricted to 15000 probes so if there were more probes in a dataset the top 15000 with the highest variance of their expression profiles were selected. For each of the 11 datasets partial correlations were calculated using the R package ‘GeneNet’ and their *p*-values found using a randomization of the dataset as a null distribution. For each gene pair the 11 *p*-values were combined using Fisher’s method, their significance level assessed using a null distribution of combined *p*-values derived from a randomization of the datasets and the resulting permutation combined *p*-values fdr adjusted using the method of Benjamini-Hochberg.

### Randomisation

In order to determine a null distribution we need to randomise the data matrices. We could choose to randomise the data matrices entirely, by permuting both the rows (i.e. pair up probes between acgh and expression experiments randomly) and columns (i.e. pair up samples between acgh and expression experiments randomly), but we considered the resulting random data to be too dissimilar to the original data, leading to over-optimistic *p*-values. We required randomised data that still reflects the structure in the data. We make the assumption that in general the acgh and expression profiles in the data are not biologically correlated, so that any observed correlation is occurring by chance alone. We generate the null distribution using random pairings of acgh and expression profiles. Figure S6 in File S1 plots the distribution of Pearson correlations of a random selection of acgh and expression profiles from the EAC dataset, Fisher Z-transformed (solid line), and overlain with the theoretical distribution for the correlations between two random variables (dashed line). The distributions are similar but the distribution for random selections of acgh and expression profiles has a greater variance, so *p*-values generated using this null distribution will be larger. The assumption that the acgh/expression correlations created by the structure in the data have no biological origins will only partly be true. So using this approach may produce overly pessimistic *p*-values, erring on the side of caution. Further experimental work would be required to determine whether using a null distribution generated differently, using entirely randomised data, would be acceptable or result in too many false positive predictions of gene pair relationships.

#### Single dataset

For the EAC dataset we require the frequency with which correlations occur between randomly selected acgh and expression profiles. The procedure for doing this is as follows. Firstly we randomise the expression matrix by permuting the probe indices. Using the randomised expression matrix and the acgh matrix we calculate and record 10^6^ correlations, which constitutes the null distribution.

#### Multiple datasets

For each of the 11 datasets in the multiple dataset study we independently generated 10^6^ correlations using the acgh matrix and the expression matrix, randomised by permuting the probe indices. These were converted to correlation *p*-values. In the case of Pearson correlations this was achieved using Fishers Z transform (R function cor.test). In the case of partial correlations this was achieved using a null distribution of partial correlations generated from random datasets of the same dimensions as the experimental dataset. This gave a 10^6^×11 matrix of correlation *p*-values. For each row of the matrix we combined the 11 *p*-values using Fisher’s method, creating a vector of 10^6^ combined *p*-values, which constituted the null distribution.

### Experimental Validation

#### RNA-interference (RNAi) assays

We screened a panel of cancer cell lines to characterize the amplification and over-expression status of selected regulating genes (ERBB2, FGFR2, ARPC1A and FANCG) to validate experimentally (Figure S1 and Figure S4 in File S1). We intentionally used a panel of four targeting-siRNAs (Qiagen) to silence the expression of any potential regulating gene to reduce off-target effects, and measured the regulatory effects in terms of mRNA readout of potential target genes following effective knockdown of their respective regulating genes (Table S3 in File S2). siRNA transfection procedures were carried out with Qiagen HiPerfect transfection reagent following manufacturer’s protocols with optimized conditions (Table S4 in File S2). Briefly, appropriate amounts of siRNAs/HiPerfect mixture were diluted in serum-free culture media (Gibco) and added to each well in a 24-well plate, in two-three technical replicates, and RNA were harvested from transfected cells at 48 hours post-transfection. We then repeated the same experiments under identical conditions three more times, giving four biological replicates per assay carried out.

#### Over-expression and rescue siRNA experiments

We then tested the regulatory relationship between ERBB2-BST1 in a reciprocal experimental setting by forcing the over-expression of ERBB2 in HSC39 cells, which do not harbor amplifications of ERBB2, using a commercially available ERBB2-over-expression plasmid vector (QIAgenes Expression Kit: catalogue number EIM0042728, plasmid ID 5160249; Qiagen). We generated an empty vector from this plasmid by cutting out the gene insert from the vector backbone using two unique restriction enzymes (PacI and XhoI; New England Biolabs) and re-ligation of blunt ends following addition of blunt ends to the cut vector backbone (data not shown) to serve as a transfection control in all experiments utilizing the ERBB2-over-expression vector. Plasmid DNA transfection was carried out using Lipofectamine 2000 (Invitrogen) following manufacturer’s protocol. Briefly, 800 ng of plasmid DNA or empty vector were transfected into cells with serum-free Opti-MEM media (Gibco), and then culture media with serum were added to the cells at 6 hours post-transfection. We measured the effects of ERBB2 over-expression in cells by quantifying the RNA extracted from transfected cells at 48 hours post-transfection using qRT-PCR.

We used rescue siRNA experiments to show specificity of our RNAi assays in selectively knocking down ERBB2, hence determining the specificity of the regulatory relationship between ERBB2-BST1. We carried out rescue siRNA experiments in OE19 cells. At 5–6 hours post-plasmid DNA transfection when fresh culture media were added to the cells, we transfected the cells with the panel of four ERBB2-targeting siRNAs, and harvested the RNA from cells at 48 hours post-plasmid transfection.

#### RNA extraction

All post-transfection RNA extractions were carried out at selected harvesting time-points using Qiazol (Qiagen) according to manufacturer’s protocol. Briefly, cells were washed with phosphate-based saline buffer before 100 µl Qiazol were added to each well on a 24-well plate and cells lysed by repetitive pipetting. The quality and quantity of RNA extracted were determined using (NanoDrop Technologies).

#### Quantitative-reverse transcription-PCR

We quantified the effects of RNAi, vector-mediated over-expression and rescue siRNA experiments by measuring the post-transfection mRNA-converted cDNA levels of regulating and target genes using qRT-PCR (RotorGene6000, Qiagen). All qRT-PCR reactions were carried out using SYBR-green JumpStart Taq Ready Mix kit (Sigma-Aldrich), following manufacturer’s protocol.

#### Statistical analysis of validation experiments

Statistical significance was determined by a linear mixed model analysis using function *lme* of the mixed model package *nlme* from the R statistical software suite. The biological sample effect was considered a random effect. While treatment by either a control procedure or one of the four siRNAs was considered a fixed effect. Each treatment-sample combination was performed on two or three technical replicates. The outcome of each experiment suppressing the regulating gene was a positive value of abundance of mRNA of the target gene. In order to enable modelling by a linear model assuming normal noise and equal variances in treatment groups, a log transform was applied to the mRNA abundance values. After this transformation Bartlett’s test for nonequal variance (R function bartlett.test) and Shapiro’s test (R function shapiro.test) for normality of residuals resulted in high *p*-values for all linear models, thus providing no reason to reject the assumptions of homoscedasticity and normality of residuals. The effect of one or two outliers was quite drastic. In order to make the analysis more robust, we therefore resolved to remove outliers whose studentized residuals were more than 2.326 away from 0, which corresponds to the lower and upper one percentile of the standard normal distribution.

The effect of interest was the contrast between the control and the mean of the four siRNA treatments. All *p*-values are one-sided since we predict the direction of the effect: negative, when the regulating gene acts as suppressor; positive, when the regulating gene acts as inducer. Finally, a multiple-testing adjustment using the Benjamini-Hochberg method was applied to all the experiments performed. A result is called significant if the BH adjusted *p*-values of the mixed model analysis is 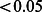 and the predicted direction of the regulating gene effect (positive or negative) was correct as judged by the fitted contrast value. These statistically significant results are marked by a double or triple star (Table 1).

**Table 1:** Results for all predicted regulatory gene interactions that were tested experimentally, single and multiple datasets combined.

Regulator	Target genes	direction	dir ok	*p*-values	out	signif	fdr	Cell line
ERBB2	BST1	+	1	0.000	1	***	0.000	OE19
	IFIT1	+	1	0.010	0	***	0.029	OE19
	PPP2R3A	+	0	0.000	1		0.000	BT474
	KCNS1	+	0	0.002	2		0.007	BT474
	PFDN5	−	1	0.000	1	***	0.000	BT474
	GAL3ST4	−	1	0.013	1	**	0.031	BT474
	PPP2R3A	+	1	0.160	1		0.213	OE19
	KCNS1	+	1	0.030	1	**	0.048	OE19
	PFDN5	−	1	0.000	1	***	0.000	OE19
	GAL3ST4	−	1	0.011	0	**	0.029	OE19
FGFR2	JAK1	+	1	0.027	2	**	0.046	HSC39
	NFIA	+	1	0.000	1	***	0.000	HSC39
	SAMD12	+	1	0.017	2	**	0.034	HSC39
ARPC1A	NCBP2	+	1	0.424	2		0.443	AsPc1
	VTI1B	+	1	0.044	1	*	0.066	AsPc1
	YEATS2	+	0	0.128	1		0.181	AsPc1
	TNFRSF8	−	1	0.017	1	**	0.034	AsPc1
	PTGDS	−	1	0.000	1	***	0.000	AsPc1
	MFNG	−	1	0.207	1		0.261	AsPc1
FANCG	KIRREL3	+	1	0.377	1		0.431	BT474
	PBX3	+	1	0.027	1	**	0.046	BT474
	CKB	−	1	0.365	1		0.431	BT474
	ALDH6A1	−	0	0.425	1		0.443	BT474
	PCDHB6	−	0	0.490	1		0.490	BT474

A total of 24 different regulating-target gene interactions were tested, of which 13 (54.2%) validated (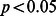).

+/−: Positive/negative gene regulations as predicted by genomic randomization (‘+’: regulating gene acts as an inducer, reduced expressions of the regulating gene lead to reduced expressions of its target genes; ‘−‘: regulating gene acts as a suppressor, reduced expressions of the regulating gene leads to increased expressions of its target genes).

dir: ‘1’ means regulation direction from validation followed genomic randomization prediction.

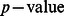
: Statistical significance according to a linear mixed model analysis.

out: Number of outliers removed for analysis.

signif: Statistical significance according to 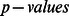 and correct directions of regulating gene effects: *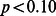, **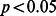, ***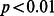.

fdr: 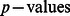 following Benhamini-Hochberg multiple-testing adjustment.

Significance of over-expression assays was determined by comparing samples treated with ERBB2-over-expression plasmid vectors to samples treated with an empty vector, analyzed using a two-tailed t-test with Mann-Whitney post-test (Prism v5.01, GraphPad Software, Inc., San Diego, CA, USA). Significance of rescue siRNA assays was determined by comparing samples treated with ERBB2-over-expression plasmid vectors, giving protective effects against targeting siRNAs, and samples treated with empty vectors, hence not protected against targeting siRNAs.

## Results

### Single Dataset: Identification of Potential Regulatory Interactions

We first tested the genomic randomization algorithm in a dataset of 54 esophageal adenocarcinoma (EAC) samples with matched aCGH and microarray gene expression data [12], [13]. We used the three conditions described above, namely i) expression of a potential regulating gene must correlate highly with its own aCGH; ii) expression of a potential target gene must correlate highly with its regulating gene’s aCGH; iii) the correlation between a regulating gene’s expression and its potential target gene’s aCGH must be low.

**Figure 2:**
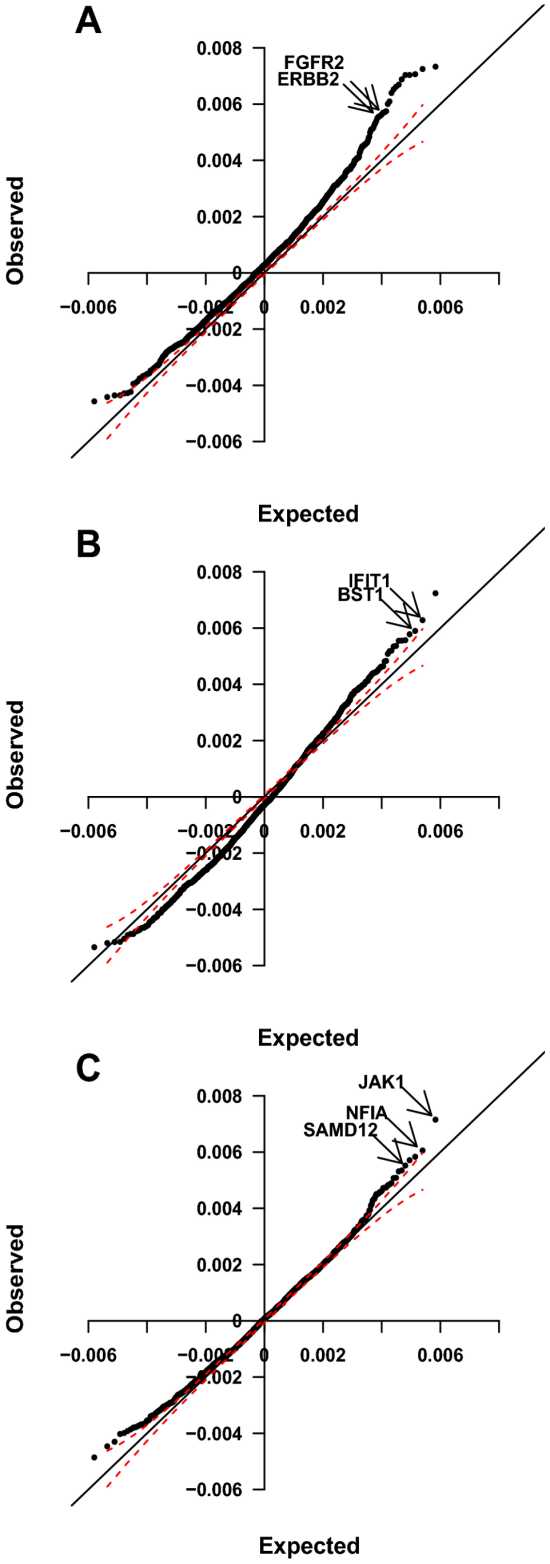
Quantile-quantile plots of observed versus expected partial correlations in the EAC dataset. (for the 2000 probes with the maximum expression variance) **A.** Partial correlations between each probe’s array comparative genomic hybridization (aCGH) profile and its own expression profile. The two potential regulating genes selected for experimental validation (ERBB2 and FGFR2) are marked in the plot. **B.** Partial correlations between ERBB2’s aCGH profile and the expression profiles of all other probes. The two genes, IFIT1 and BST1, selected for experimental validation are marked. **C.** Partial correlations between FGFR2’s aCGH profile and the expression profiles of all other probes. The three genes, JAK1, NFIA and SAMD12, selected for validation are marked. In all plots 5% confidence intervals are marked by dashed lines.

Using the significance of the first of the three conditions, the correlations between expression and aCGH, we identified potential regulating genes (Figure 2A and Table S2 in File S2, and Sheet S1 in Results S1). There were 28 genes with 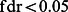 and 61 with 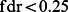; fdr being the false discovery rate indicated by the Benjamini-Hochberg adjusted 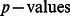. We selected two potential regulating genes for experimental validation, namely ERBB2 (v-erb-b2 erythroblastic leukemia viral oncogene homolog 2) and FGFR2 (fibroblast growth factor receptor 2). These were selected based on the statistical significance of the correlations (ERBB2: 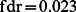, FGFR2: 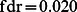) and also because of their role as receptors in signalling pathways and their biological importance as both have known roles in carcinogenesis.

We used the second condition, the correlation between the expression of a potential target gene and its regulating gene’s aCGH, to predict target genes for ERBB2 and FGFR2 in turn. The resulting lists (Table 2 and Sheets S2–S5 in Results S1) were filtered according to the third condition, namely that the correlation between a regulating gene’s expression and its target gene’s aCGH must be low. For ERBB2 there were 19 genes positively correlated and 11 negatively correlated with 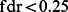. For FGFR2 there were 12 genes positively correlated and 0 negatively correlated with 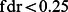. The highest ranked predicted target genes were selected for experimental validation, namely ERBB2-BST1, ERBB2-IFIT1, FGFR2-JAK1, FGFR2-NFIA and FGFR2-SAMD12 (Figure 2B–C and Table 2).

**Table 2:** Single dataset: List of top 10 potential target genes whose expression was highly correlated with the aCGH status of regulating genes, ERBB2 and FGFR2.

Regulating gene	Chrom	Target gene	Chrom	fdr	sign
ERBB2	17	IFIT1	10	0.024	+
		BST1	4	0.049	+
		SLCO1B3	12	0.053	+
		PPARGC1A	4	0.058	+
		ALPPL2	2	0.074	+
		PRRX2	9	0.092	+
		MSX2	5	0.104	+
		RHOU	1	0.161	+
		GSTM3	1	0.200	+
		ATP10B	5	0.200	+
FGFR2	10	JAK1	1	0.003	+
		NFIA	1	0.044	+
		SAMD12	8	0.082	+
		DCUN1D1	3	0.100	+
		DSG1	18	0.136	+
		PNLIPRP2	10	0.180	+
		PTPN2	18	0.180	+
		DRD5	4	0.180	+
		CKMT2	5	0.204	+
		MCART1	9	0.204	+

Based on the false discovery rate (fdr), which is the Benjamini-Hochberg adjusted 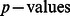. The sign of the correlation (positive or negative, +/−) is indicated. Hypothetical proteins have been excluded from the list. The top three gene pairs were selected for subsequent experimental validations; except for ERBB2-SLCO1B3 because primers designed for SLCO1B3 could not be optimized for qRT-PCR assays. Chrom 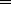 Chromosome.

### Single Dataset: Experimental Validation of Predicted Interactions

For experimental validation, we selected cancer cell lines that harbor amplifications of ERBB2 (OE19) and FGFR2 (HSC39), and used cell lines without these amplifications as controls (Figure S1 in File S1). This allowed us to test whether gene amplifications and over-expression of regulating genes were indeed the driving force behind the observed regulatory interactions between genes. To increase the stringency and reduce off-target effects in these validation tests, we used a panel of four different gene-targeting siRNAs in each RNAi assay and carried out each identical experiment (biological replicate) at least three times with appropriate technical replicates each time. In addition, for a selected gene pair, vector-mediated over-expression and siRNA rescue experiments were performed to demonstrate the regulatory relationship between the regulating gene and its target gene. The experimental readout for all validation assays was mRNA expression level changes measured using qRT-PCR assays.

In data interpretation, a positive regulation predicted by genomic randomization means a successful validation assay must show reduced gene expression of potential target genes following silencing of their regulating gene with an overall statistical significance (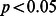), and vice versa. In the following we report adjusted 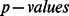 of a mixed effects analysis of variance (ANOVA) test of the contrast between the logarithm of mRNA values in the untreated control and the four treatments by siRNAs. Treatment effects were assumed fixed and biological sample effects as random.

**Figure 3:**
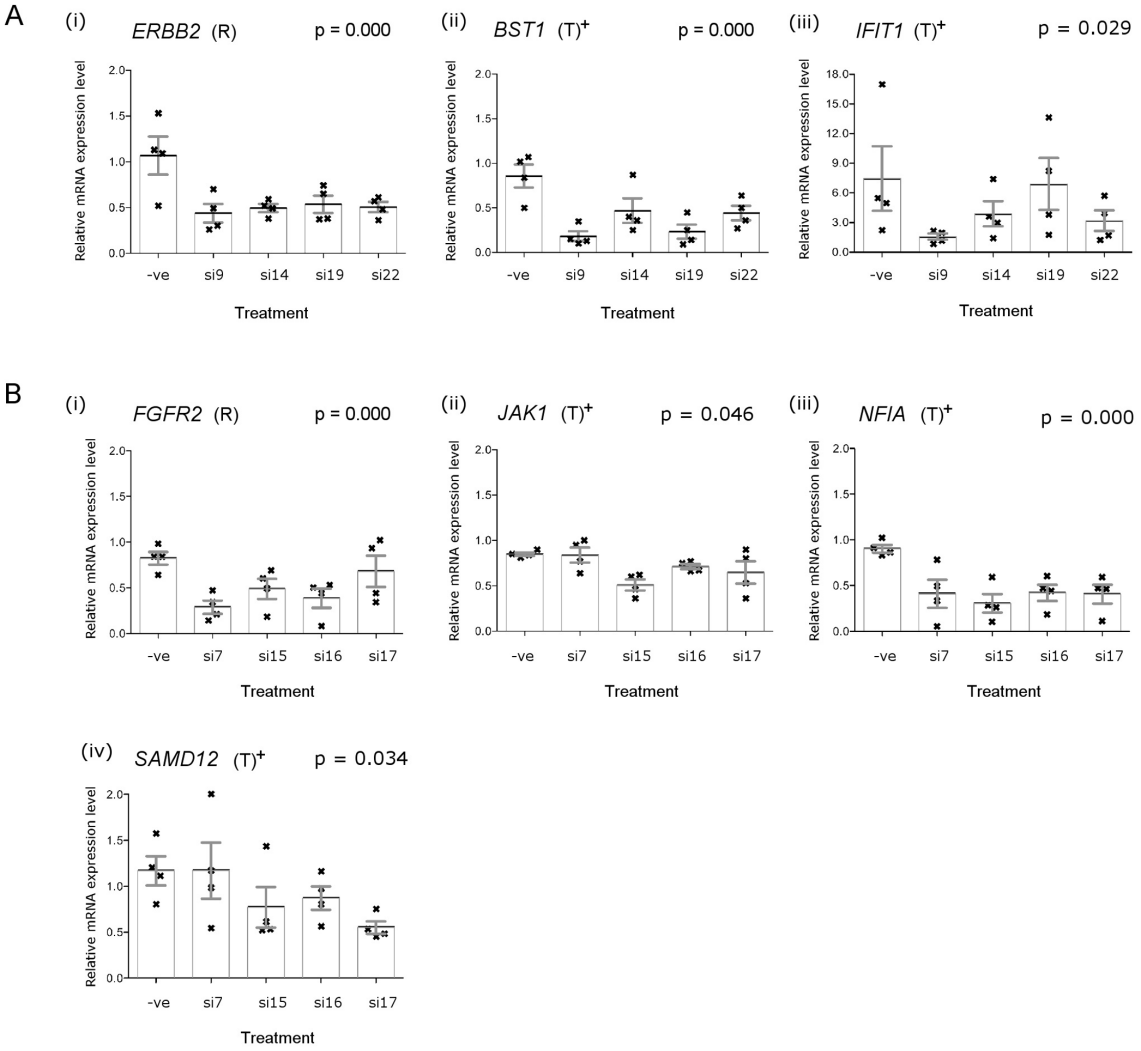
qRT-PCR quantifications of mRNA levels in RNA interference assays in selected cancer cell lines with amplifications of regulating genes. (**a**) Silencing of ERBB2 in OE19 cells, which harbor ERBB2 amplifications, showed significant reduction of the mRNA levels of: (i) ERBB2 and its predicted target gene, (ii) BST1 (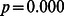) and (iii) IFIT1 (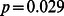). (**b**) Silencing of FGFR2 in HSC39 cells, which harbor FGFR2 amplifications, showed significant reduction of the mRNA levels of: (i) FGFR2 and its predicted target genes, (i) JAK1 (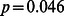), (ii) NFIA (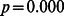) and (iii) SAMD12 (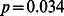). Note: (R) regulating genes targeted by targeting siRNAs; (T) potential target genes tested, predicted to be positively-regulated (+) by regulating genes; (−ve) non-silencing negative siRNAs. siRNAs used in the panel are named according to their commercial product name (Qiagen). The vertical-axes for all plots are fixed from 0.0–2.0, except for the plot for IFIT1, where the vertical-axis is customized due to the variability in the gene expression changes.

Matching our predictions, RNAi assays demonstrated that siRNA-mediated ERBB2 silencing led to significant reduction in expression levels of bone marrow stromal cell antigen I (BST1; 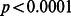) and interferon-induced protein with tetratricopeptide repeats 1 (IFIT1; 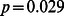). siRNA-mediated FGFR2 silencing led to reduced expression levels of Janus kinase I (JAK1; 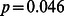), nuclear factor I/A (NFIA; 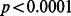) and sterile alpha motif domain containing 12 (SAMD12; 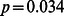) (Figure 3). Silencing of these regulating genes showed no effect in cell lines without amplifications of ERBB2 and FGFR2 (see Figure S2 in File S1).

In a reciprocal experimental setup, we observed elevated mRNA expression levels of ERBB2 and BST1 in HSC39 cells, which do not have ERBB2 amplifications and over-expression, following transfection with ERBB2-over-expression vector (Figure S3a in File S1). We further proved the specificity of siRNA-mediated ERBB2 silencing with siRNA rescue experiments, in which OE19 cells were protected from ERBB2-targeting siRNAs through vector-mediated ERBB2 over-expression, leading to no reduction in expression levels of either ERBB2 or BST1 (Figure S3b in File S1). These results thus far validated all five (100%) of the predicted regulatory gene interactions brought forward for experimental testing, suggesting the potential of the genomic randomization algorithm in identifying novel functional gene relationships. The results are contained in Table 1.

### Multiple Datasets: Identification of Potential Regulatory Interactions

Having demonstrated the ability of the technique to identify gene interactions in esophageal adenocarcinoma genomes, we obtained publicly available copy number and gene expression data from nine other studies (Table S1 in File S2) to add to the EAC dataset to further test the applicability of the approach. Combined analysis of these ten cancer datasets using genomic randomization generated a list of 1,410 potential regulating genes (Table 3 and Sheet S6 in Results S1). We selected the three highest ranked potential regulating genes from the list in Table 3 for which there are known cell lines containing amplifications of these genes (CONAN - Cancer Genome Project, Wellcome Trust Sanger Institute: http://www.sanger.ac.uk/cgi-bin/genetics/CGP/conan/search.cgi); namely ERBB2 (v-erb-b2 erythroblastic leukemia viral oncogene homolog 2), ARPC1A (actin related protein 2/3 complex subunit 1A) and FANCG (Fanconi anemia complementation group G).

**Table 3:** Multiple datasets: List of potential regulating genes arranged according to the number of datasets in which the gene has significant aCGH-expression correlations, and according to the fdr adjusted 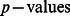, derived from 11 individual correlation 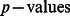.

Regulatinggene	Chromosome	fdr	Number of datasets†
ERBB2*	17	0.00004	9
GRB7	17	0.00004	8
ARPC1A*	7	0.00004	7
STIP1	11	0.0002	7
FANCG*	9	0.0005	7
RBM6	3	0.0006	7
RAD23B	9	0.0006	7
SRRM2	16	0.0006	7
PPFIA1	11	0.00004	6
PEX1	7	0.00004	6

† Number of datasets contributing to the significance of each correlation was important to ensure that no single cohort or cancer type was introducing bias to the analysis.

* Regulating genes selected for subsequent validation assays via RNAi experiments. Cell lines with amplifications of these genes were reported (CONAN - Cancer Genome Project, Wellcome Trust Sanger Institute: http://www.sanger.ac.uk/cgi-bin/genetics/CGP/conan/search.cgi). GRB7 was not chosen because of its proximity with ERBB2.

We then identified potential target genes for each of these potential regulating genes using genomic randomization (Table 4 and Sheets S7–S12 in Results S1). The number of genes with fdr adjusted 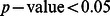, involved in more than 4 datasets and on different chromosomes to their potential regulating gene (hence potential *trans*-acting regulations) are 25 for ERBB2/positive, 27 for ERBB2/negative, 2 for ARPC1A/positive, 13 for ARPC1A/negative, 0 for FANCG/positive and 2 for FANCG/negative. Fifteen potential regulating-target pairs were selected for experimental validation (see Table 4). These were the highest ranked *trans*-acting regulating-target pairs, excluding those target genes for which primers could not be optimised for the qRT-PCR assays (note: on different chromosomes, hence potential *trans*-acting regulations, except for one pair, FANCG-PBX3). Both positive and negative regulations were included to serve as reciprocal experimental controls.

**Table 4:** Multiple datasets: List of top 10 potential target genes (5 positive correlations and 5 negative correlations) for the three potential regulating genes selected for experimental validation: ERBB2, ARPC1A and FANCG.

Regulating gene	Chrom	Target gene	Chrom	Num	fdr
ERBB2/positive	17	PPP2R3A*	3	6	0.0019
		GAS2	11	5	0.0012
		KCNS1*	20	5	0.0022
		PTPN11	12	4	0.0008
		SRPK1	6	4	0.0010
ERBB2/negative	17	PFDN5*	12	6	0.0005
		GAL3ST4*	7	5	0.0092
		OLFML3	1	5	0.0107
		ARL3	10	4	0.0014
		COX7A1	19	4	0.0016
ARPC1A/positive	7	NCBP2*	3	4	0.0401
		VTI1B*	14	4	0.0413
		GTF3C3	2	3	0.0225
		YEATS2*	3	3	0.0321
		SPTBN2	11	3	0.0347
ARPC1A/negative	7	TNFRSF8*	1	5	0.0017
		PTGDS*	9	5	0.0038
		MFNG*	22	5	0.0112
		IL16	15	4	0.0076
		TGFBR2	3	4	0.0080
FANCG/positive	9	CTLA4	2	4	0.0800
		KIRREL3*	11	3	0.0076
		PBX3*	9	3	0.0124
		AGTR2	X	3	0.0137
		GLRA2	X	3	0.0172
FANCG/negative	9	CKB*	14	4	0.0104
		ALDH6A1*	14	4	0.0250
		PCDHB6*	5	3	0.0158
		CRYGD	2	3	0.0294
		HADHA	2	3	0.0510

Target genes are arranged according to the number of datasets (Num) in which the gene pair has significant aCGH-expression correlations, and the fdr adjusted combined 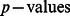. Target genes located on the same chromosome as their potential regulating genes are excluded from the lists. Asterisks (*) mark the potential target genes investigated in validation experiments. Chrom = Chromosome.

### Multiple Datasets: Experimental Validation of Predicted Interactions

**Figure 4:**
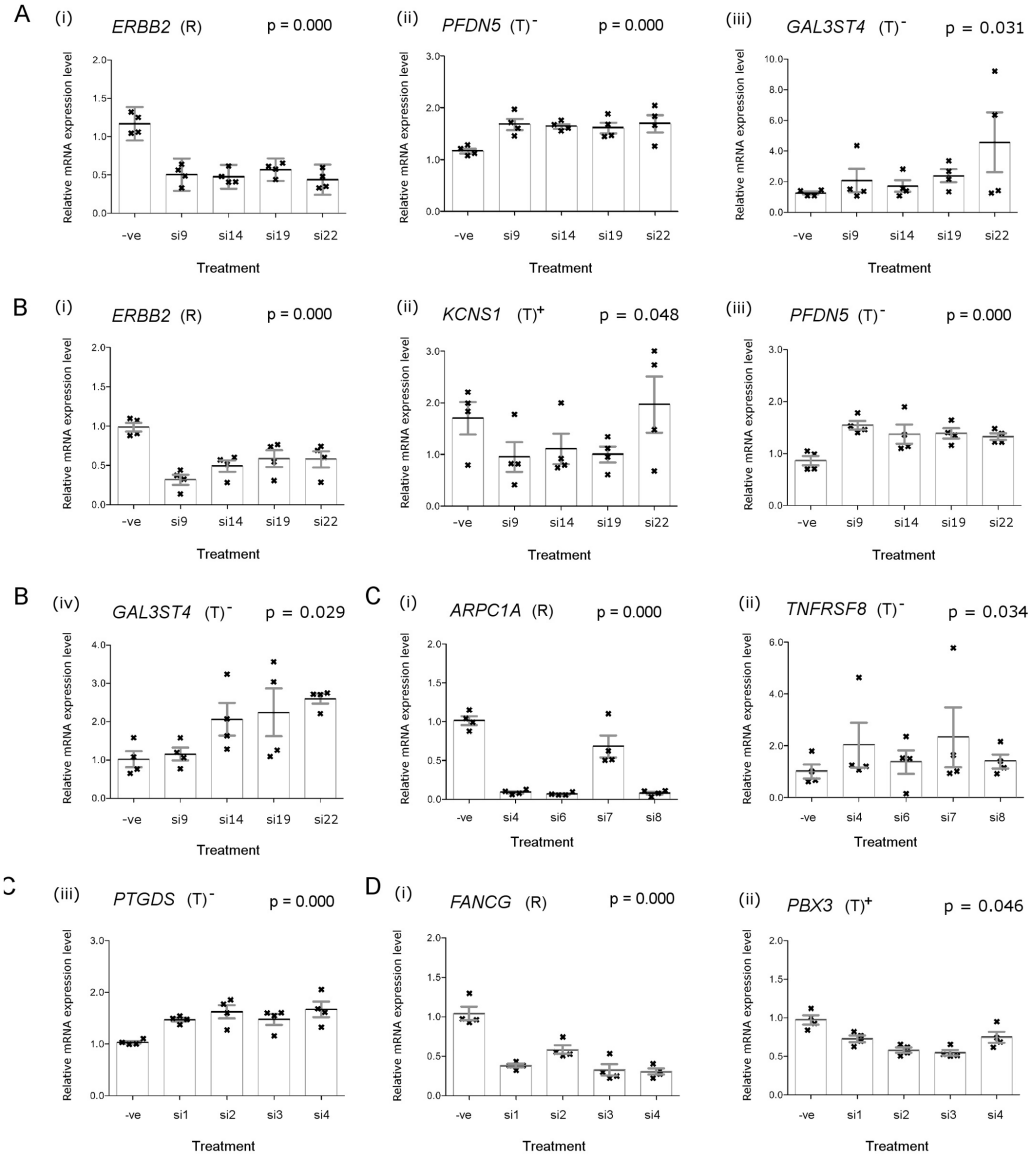
qRT-PCR quantifications of mRNA levels following RNA interference assays in selected cancer cell lines with amplifications of regulating genes. (**a**) Effects of ERBB2-targeting siRNAs treatment in BT474 cells, showing: (i) effective silencing of ERBB2 (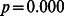); leading to significant up-regulations of (ii) PFDN5 (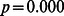) and (iii) GAL3ST4 (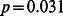). (**b**) Effects of ERBB2-targeting siRNAs treatment in OE19 cells, showing: (i) effective silencing of ERBB2 (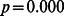); leading to (ii) down-regulation of KCNS1 (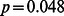; (iii) up-regulation of PFDN5 (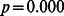) and (iv) up-regulation of GAL3ST4 (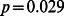). (**c**) Effects of ARPC1A-targeting siRNAs treatment in AsPc1 cells, showing: (i) effective silencing of ARPC1A (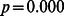); leading to significant up-regulations of (ii) TNFRSF8 (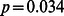) and (iii) PTGDS (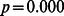). (**d**) Effects of FANCG-targeting siRNAs treatment in BT474 cells, showing: (i) effective silencing of FANCG (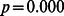); leading to significant up-regulation of (ii) PBX3 (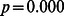). Note: (R) regulating genes targeted by targeting siRNAs; (T) potential target genes, positively- (+) or negatively-regulated (−) by regulating genes; (−ve) non-silencing negative siRNAs; siRNAs used in the panel are named according to their commercial product name (Qiagen). The vertical-axes for plots showing silencing of regulating genes are fixed from 0.0–2.0 whilst the vertical-axes for plots of target genes were customized according to the variability in the gene expression levels.

Using a similar RNAi experimental setting as before, we performed a thorough investigative validation to verify relationships between predicted regulating-target pairs in cell lines with amplification of ERBB2, ARPC1A or FANCG (Figure S4 in File S1). siRNA-mediated ERBB2 silencing was performed in two cell lines that harbor ERBB2 amplifications and over-expression, BT474 and OE19 cell lines. ERBB2 silencing in these cells led to significant up-regulation of prefoldin subunit 5 (PFDN5; 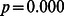) and galactose-3-O-sulfotransferase-4 (GAL3ST4; 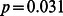) expression levels in both cell types. In addition, reduced expression of potassium voltage-gated channel, delayed rectifier, subfamily S, subunit 1 (KCNS1) was observed in OE19 cells (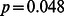), though not seen in BT474 cells (Figure 4A–B). In the pancreatic AsPc1 cell line, siRNA-mediated ARPC1A silencing led to significant up-regulation of tumor necrosis factor receptor superfamily, member 8 (TNFRSF8; 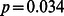) and prostaglandin D2 synthase 21 kDa (PTGDS; 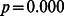) (Figure 4C). Lastly, regulatory relationships of predicted target genes with FANCG were tested in breast BT474 cell line, showing that a predicted target gene, pre-B-cell leukemia homeobox 3 (PBX3; 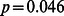) was affected by FANCG silencing (Figure 4D). The qRT-PCR results for the gene pairs which did not validate are shown in Figure S5 in File S1. The results are contained in Table 1.

### Comparing Pearson and Partial Correlation Analyses

For the multiple datasets, the gene pairs that were tested experimentally were predicted by a Pearson correlation analysis. For comparison we retrospectively ran a partial correlation analysis on the multiple datasets for these gene pairs (see Methods section for details). Conversely, the gene pairs that were tested experimentally from the single esophageal cancer dataset were predicted from a partial correlation analysis, so for comparison we also ran a Pearson correlation analysis on this dataset. In this way we could compare the predictions made by a partial correlation analysis and a Pearson correlation analysis in light of the results from the 24 validation experiments. The results of this comparison are given in Table 5. We found that for Pearson correlation (with fdr 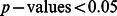), 9 of the 24 validation experiments were predicted correctly (i.e. 9 true positives or true negatives). The partial correlation analysis however predicted 16 of the 24 validation results correctly, a success rate of 66%. This suggests that using partial correlations increases the accuracy of the method by an amount that warrants the additional computational complexity.

**Table 5:** Comparing the false discovery rate (fdr) adjusted 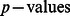 obtained from an analysis of both datasets using Pearson correlation and using partial correlation, and the performance of their predictions in light of the results from the 24 validation experiments.

Genes		Experiment	Pearson		Partial	
Regulator	Target	Fdr	fdr	performance	fdr	performance
ERBB2	BST1*	0.000	0.329	FN	*0.024*, 0.288	*TP*, FN
	IFIT1*	0.010	0.245	FN	*0.049*, 0.015	*TP*, TP
	PPP2R3A	(0.000)	0.002	FP	0.018	FP
	KCNS1	(0.002)	0.002	FP	0.002	FP
	PFDN5	0.000	0.001	TP	0.005	TP
	GAL3ST4	0.013	0.009	TP	0.021	TP
	PPP2R3A	0.160	0.002	FP	0.018	FP
	KCNS1	0.030	0.002	TP	0.002	TP
	PFDN5	0.000	0.001	TP	0.005	TP
	GAL3ST4	0.011	0.009	TP	0.021	TP
FGFR2	JAK1*	0.027	0.937	FN	*0.003*, 0.014	*TP*, TP
	NFIA*	0.000	0.937	FN	*0.044*, 0.300	*TP*, FN
	SAMD12*	0.017	0.937	FN	*0.082*, 0.029	*FN*, TP
ARPC1A	NCBP2	0.424	0.040	FP	0.587	TN
	VTI1B	0.044	0.041	TP	0.757	FN
	YEATS2	(0.128)	0.032	FP	0.641	TN
	TNFRSF8	0.017	0.002	TP	0.001	TP
	PTGDS	0.000	0.004	TP	0.045	TP
	MFNG	0.207	0.011	FP	0.1	TN
FANCG	KIRREL3	0.377	0.008	FP	0.016	FP
	PBX3	0.027	0.012	TP	0.005	TP
	CKB	0.365	0.010	FP	0.083	TN
	ALDH6A1	(0.425)	0.025	FP	0.102	TN
	PCDHB6	(0.490)	0.016	FP	0.014	FP

TP = True Positive, TN = True Negative, FP = False Positive, FN = False Negative, based on a fdr 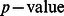 threshold of 0.05. () = experimental direction of change does not agree with prediction.

* predictions from the single dataset. Partial correlations in italics were calculated using a 2000 probe subset of the single dataset, otherwise partial correlations were calculated from the multiple datasets using up to 15000 probes.

## Discussion

In this paper we propose a new approach to predicting regulating genes and their targets from matched aCGH and expression data on cancer samples and we test our predictions rigorously using gene silencing, over-expression and qRT-PCR. Our experimental evidence validated 13/24 (54.2%) of regulating-target gene interactions tested based on genomic randomization predictions at an adjusted significance level of 0.05. These included 5/24 (21%) highly statistically significant and novel regulations (adjusted 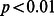). Overall, 14/24 (58.3%) could be deemed as highly possible functional interactions across a number of cancer types (adjusted 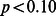) (Table 1). For ERBB2 in particular, 5/6 (83.3%; adjusted 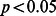) predicted target genes were demonstrated to be influenced by ERBB2 expression levels. Our initial analysis used partial correlation analysis on the single dataset and, for computational reasons, Pearson correlations on the multiple dataset analysis. A retrospective analysis suggests that partial correlations predict interactions better than Pearson correlations.

The sensitivity and specificity are for the experimentally validated gene pairs, which were the top ranked gene pairs. Further work will be required to determine what the sensitivity and specificity would be for genes ranked lower down in the lists. Leek et al. [16] present a statistical approach to this type of problem that reduces the amount of further experimentation required.

The lack of statistical significance in the validation of some regulating-target gene interactions that were predicted by the analysis could be due to more than one gene being involved in the regulation of the selected target genes, or due to differences between cell lines and cellular physiological conditions. In general the correlation of a predicted regulating-target gene interaction was significant in only some of the datasets used and entirely uncorrelated in the remaining, indicating the importance of tissue type in these gene regulatory relationships, and strongly suggesting that the selection of cell line of appropriate tissue origins and physiological conditions are key to successful validation. This is an aspect of the technique that requires further investigation and experimentation. If two or more potential regulating genes are always amplified together, and by the same amount, in every sample of a dataset then it will of course be impossible to separate the potential effects of these co-amplified genes.

Fisher’s method assumes independence of 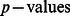. In practice we find that if a regulator-target pair has a high correlation in one dataset it does not mean that the pair will necessarily have a high correlation in any other dataset, so there is some degree of independence. However there is likely to be some dependence which may result in overly optimistic 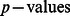. In contrast, the randomisation method adopted for generating null distributions was conservative.

In contrast to other methods of integrating aCGH and expression data for the prediction of driver genes (for example, [11]), we use aCGH data also to circumvent the problem of causal confounding that arises whenever prediction of regulatory relationships is based solely on expression correlation. Hence our approach fully utilizes the genomic randomization provided naturally in cancer samples to predict regulatory relationships.

Since the genes for which a regulatory network can be derived are all, by definition, affected by copy number variation, they almost certainly contain many key players involved in the disease process itself. The regulatory gene pairs we validated show a range of potential in terms of their novelty and known biological functions. For example, FGFR2-JAK1 relationship has been previously demonstrated from a purely experimental paradigm, where signal transducer and activator of transcription 3 (STAT3) was shown to bind to FGFR2 [17].

ERBB2 is an important receptor tyrosine kinase in cancer and amplification of this locus has been identified as a therapeutic target in cancers such as breast and gastric adenocarcinoma [18], [19]. It codes for a membrane bound receptor tyrosin kinase of the erbB growth factor family, comprising ERBB1, ERBB2, ERBB3, and ERBB4. Since it is the preferred dimerisation partner of the other members of the family, a higher concentration of ERBB2 protein probably increases EGFR signalling. This signal transduction chain leads into several well known pathways such as the mitogen-activated protein kinase (MAPK), the phosphoinositide 3-kinase (PI3K/Akt), or the signal transducer and activator of transcription (STAT) pathway. Overall EGF signalling promotes cell growth and division and suppresses apoptosis. The transcriptional target genes in these pathways are reasonably well characterised. However, as our analysis shows, the strongest effect of increased or decreased ERBB2 concentration is seen on genes outside these pathways.

Search of known signalling pathways involving ERBB2 shows no intersection with target genes found in our approach. However, this is mainly due to the very generic character of signalling pathways as available in the literature. Our approach therefore constitutes an important complementary step to reveal the biological function of genes such as ERBB2. Knowledge gained from our analyses could be used as a way to delineate how potential target genes are linked to one another and to their regulating genes in a ‘gene interactome network’ map. Our genomic randomization prediction coupled with experimental evidence suggests that BST1, PFDN5, KCNS1 and GAL3ST4 are novel downstream targets of ERBB2 and hence the EGF signalling pathway. Of these, PFDN5 is interesting because of its role in repressing the transcriptional activity of proto-oncogene v-myc myelocytomatosis viral oncogene homolog (MYC) [20], [21]. BST1, recently reported to be part of a risk locus for Parkinson’s disease via genome-wide association study, might be another interesting target due to its role in B cell growth regulation [22]. Besides, KCNS1 and GAL3ST4 could be functionally important due to their respective roles as an ion channel and an enzyme that catalyzes the process of protein glycosylation.

The third potential regulating gene validated in this study was ARPC1A. Being a cytoskeletal protein, ARPC1A has been reported to be involved in the regulation of cell migration and invasion in pancreatic cancer [23]. Interestingly, ARPC1A has also been reported to be regulated by the EGF signaling pathway [24]. None of the predicted target genes of ARPC1A have been previously reported in the context of cancer. Other biologically interesting targets include PBX3 and FANCG. PBX3 has been reported to be over-expressed in prostate cancer [25], whilst FANCG belongs to a group of proteins known as the Fanconi anemia complementation family and has been reported to be important in DNA damage repair pathways [26].

In this study we chose four potential regulating genes and their predicted target genes for experimental validation due to time and reagent constraints. There are other promising regulating-target gene predictions listed in Tables 3 and 4. For example, stress-induced phosphoprotein 1 (STIP1), an adaptor protein that coordinates the assembly of heat shock proteins, has been reported to be over-expressed in colon and pancreatic cancers [27]. A recent study reported the association of STIP1 with tumour invasion in pancreatic cancer using RNAi silencing [28], highlighting STIP1 as a potential cancer gene. Other regulating genes identified that could play a role in carcinogenesis include the RNA binding motif protein 6 (RBM6), which has been identified as part of a novel fusion gene in acute megakaryoblastic leukemia [29]; and protein tyrosine phosphatase, receptor type, f polypeptide (PTPRF), interacting protein (liprin), alpha 1 (PPFIA1), which is amplified in up to 15% of breast cancer and frequently co-amplified with cyclin D1 (CCND1) [30].

We demonstrated regulatory relationships for individual pairs using qRT-PCR that enabled us to test the predictions in specifically designed experiments, which we deemed much more robust than validation by gene enrichment analysis as reported in other studies [11]. Results suggest partial correlation analysis is superior to Pearson correlation. The level of experimental validation suggests this computational approach is a useful additional tool in the search for evidence of gene regulatory relationships. The percentage of true positives is high enough for the results to be useful as suggestions for experimental investigation or to be included with other evidence in probabilistic models of gene regulatory networks.

## Supporting Information

Code S1 **R analysis code.**
(R) Click here for additional data file.

Data S1 **Example data.**
(RDATA)

File S1 **Supplementary Figures S1 to S6.**
(PDF)

File S2 **Supplementary Tables S1 to S4.**
(PDF)

Results S1 **Complete results lists from the single dataset analysis and the multiple dataset analysis.**
(XLS)
