# Supplementary material for: Testing the Utility of an Integrated Analysis of Copy Number and Transcriptomics Datasets for Inferring Gene Regulatory Relationships
Source: PLoS One. 2013 May 30;8(5):e63780. doi: 10.1371/journal.pone.0063780 (PMC3667814; doi:10.1371/journal.pone.0063780)

Supplementary Figure S1

Screening for cell lines that harbor amplifications of ERBB2 and FGFR2. Cell types: Het1A, Nes (squamous esophageal epithelium); HSC39, MKN45 (diffuse type gastric carcinoma); OE33, FLO, OE19, OC3 (esophageal adenocarcinoma); KYSE410 (esophageal squamous cell carcinoma); HUP-T4, AsPc1 (pancreatic adenocarcinoma); BT474 (breast ductal carcinoma). DNA and RNA were extracted from a panel of 12 cell lines, derived from different tissue types. Quantification of DNA and RNA levels of ERBB2 and FGFR2 were carried out using qRT-PCR assays. Cell lines with elevated levels of DNA (black bars) and RNA (grey bars) were then chosen for subsequent RNAi and/or siRNA rescue experiments. Relative gene expression levels of genes of interest were normalized to that of the lowest expressing cell lines, i.e. ERBB2 expressions were normalized to the level in Het1A cell line whilst FGFR2 expressions were normalized to that of OE19. Based on these results, OE19 cells were selected for ERBB2-targeted RNAi experiments and HSC39 cells were selected as a control cell line that do not harbor ERBB2 amplifications. Similarly, HSC39 cells were selected for FGFR2-targeting RNAi experiments and OE19 cells were selected as its control cell line.

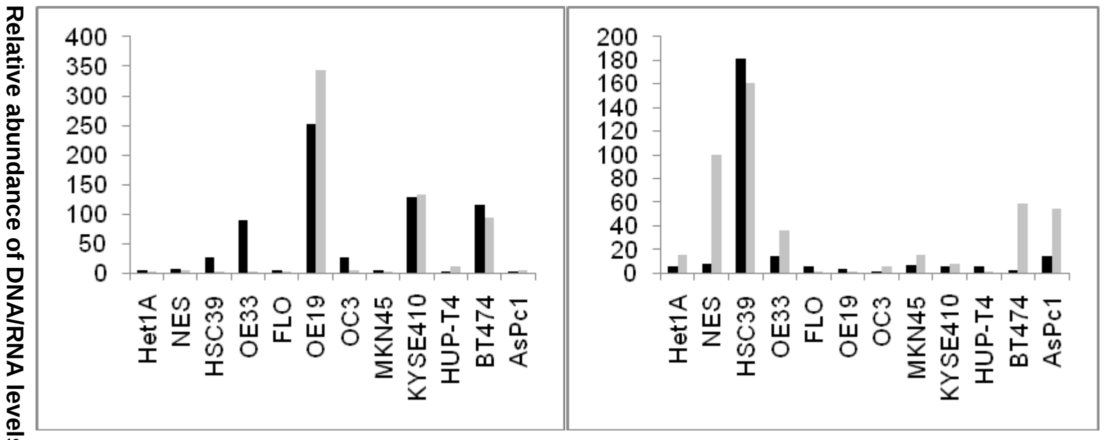

Cell line panel – *ERBB2* **Cell line panel – *FGFR2***

Supplementary Figure S2

qRT-PCR quantification of mRNA levels of genes of interest in RNA interference (RNAi) experiments. (i-iii) *ERBB2*-targeting RNAi assays in HSC39 cells, which do not harbor *ERBB2* amplifications, showing no significant changes in mRNA expression levels of *BST1* and *IFIT1* following effective silencing of *ERBB2*. (iv-vii) *FGFR2*-targeting RNAi assays in OE19 cells, which do not harbor *FGFR2* amplifications, showing no significant changes in mRNA expression levels of *JAK1*, *NFIA* and *SAMD12* following effective silencing of *FGFR2*.

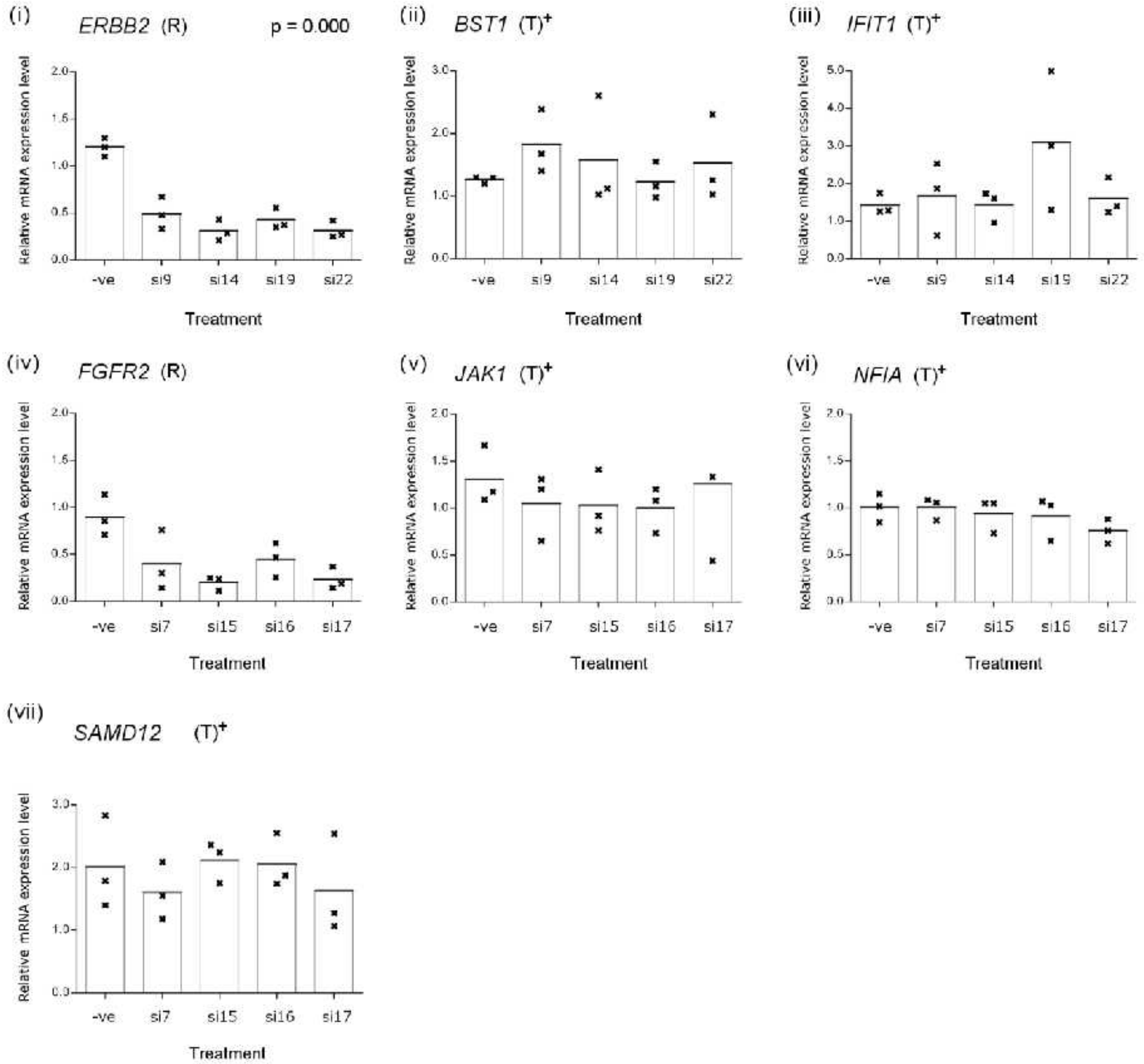

Data are results of three independent biological replicates and are represented as median and value ranges. (R) regulator genes targeted by targeting siRNAs; (T) potential target genes, positively- (+) or negatively-regulated (-) by regulator genes; (-ve) non-silencing negative siRNAs; p-values for non-significant validation are not shown.

Supplementary Figure S3

qRT-PCR quantifications of mRNA levels in vector-mediated over-expression and rescue siRNA experiments. A) Vector-mediated *ERBB2* over-expression assays in HSC39 cells, which do not harbor *ERBB2* amplifications. (i-ii) Transfection of HSC39 cells with *ERBB2*-expressing plasmid vectors (V) led to significant increase in mRNA levels of *ERBB2* itself and its target gene, *BST1* (p=0.0286), compared to cells treated with an empty vector (EV). B) Rescue siRNA assays in OE19 cells. (i-ii) Restoration of mRNA levels of *ERBB2* and *BST1* in OE19 cells treated with *ERBB2*-targeting siRNAs following transfection with either *ERBB2*-expressing plasmids (+) or empty vectors (-). (iii-iv) siRNA-14 (si14) acted as an internal control for rescue siRNA assays as it targets a sequence present within the *ERBB2*-expressing plasmid used, therefore was not able to protect against *ERBB2* silencing. Data are results of four independent biological replicates and are represented as mean ± SEM (bar). * $p < 0.05$; ** $p < 0.01$; *** $p < 0.001$; ns: $p > 0.05$. Individual data points (crosses) represent median values of three technical replicates from each biological replicate experiment. siRNAs used in the panel are named according to their commercial product name (Qiagen).

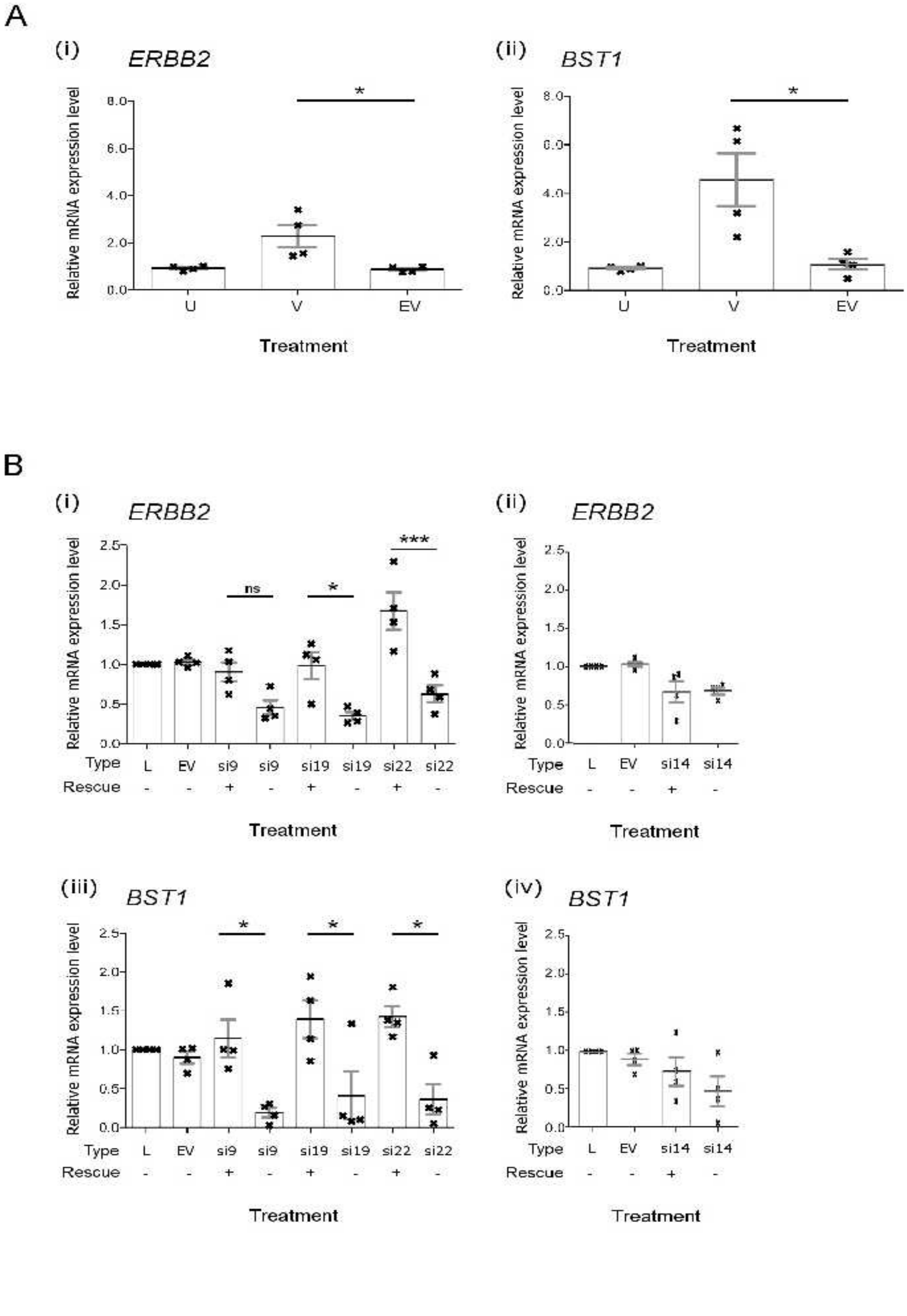

Testing the utility of an integrated analysis of copy number and transcriptomics datasets for inferring gene regulatory relationships *Xin Yi Goh, Richard Newton, Lorenz Wernisch, Rebecca C Fitzgerald*

Supplementary Figure S4

Cell line screens to identify cell lines that harbor amplifications of potential regulator genes, *ARPC1A* and *FANCG*, for RNA-interference experiments. DNA and RNA were extracted from a panel of 12 cell lines, consisting of different tissue types. Quantifications of DNA and RNA levels of *ARPC1A* and *FANCG* were carried out using qRT-PCR assays. Cell lines with elevated levels of DNA (black bars) and RNA (grey bars) were then chosen for subsequent RNAi experiments. Relative gene expressions of genes of interest were normalized to the level present in the lowest expressing cell lines, i.e. *ARPC1A* expressions were normalized to the level in OE19 cell line whilst *FANCG* expressions were normalized to that of MKN45.

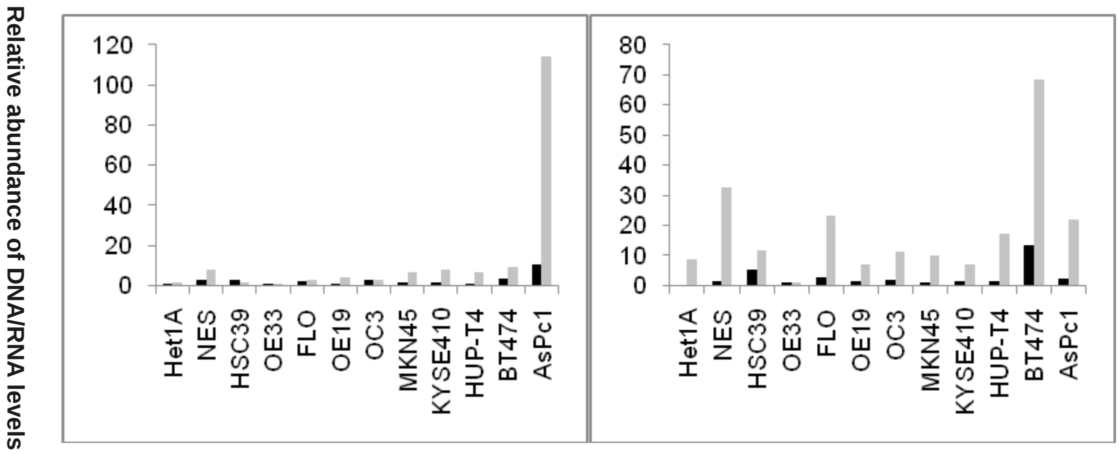

Cell line panel – *ARPC1A* **Cell line panel – *FANCG***

Cell types: Het1A, Nes (squamous esophageal epithelium); HSC39, MKN45 (diffuse type gastric carcinoma); OE33, FLO, OE19, OC3 (esophageal adenocarcinoma); KYSE410 (esophageal squamous cell carcinoma); HUP-T4, AsPc1 (pancreatic adenocarcinoma); BT474 (breast ductal carcinoma).

Based on these results (and *ERBB2* screens as shown in Figure 3), AsPc1 cells were selected for *ARPC1A*-targeting RNAi experiments and similarly, BT474 cells were selected for *FANCG*-targeting RNAi experiments, OE19 and BT474 cells were selected for *ERBB2*-targeting RNAi experiments. Control cells lines were not utilized for these experiments because having reciprocal effects (positive and negative regulations by potential regulator genes) upon targeting siRNA treatments served as internal controls.

Supplementary Figure S5

qRT-PCR quantification of mRNA levels of predicted target genes in RNAi assays, of which statistical significance in the gene-gene regulation had not been achieved. a) *ERBB2*-targeting siRNA assays in BT-474 cells, which harbor *ERBB2* amplifications, showing: (i-ii) up-regulations of *GAL3ST4* and *KCNS1* mRNA expression levels upon effective silencing of *ERBB2*. Although these two interactions were statistically significant, they were discounted because both *PPP2R3A* and *KCNS1* had been predicted by genomic randomization to be positively regulated by *ERBB2*, which means their mRNA expression levels should decrease following silencing of *ERBB2*. b) *ERBB2*-targeting siRNA assays in OE19 cells, which harbor *ERBB2* amplifications, showing: (i) no significant changes in the mRNA expression level of *PPP2R3A*. c) *ARPC1A*-targeting siRNA assays in AsPc-1 cells, which harbor *ARPC1A* amplifications, showing no significant changes in the mRNA expression levels of: (i) *NCBP2*, (ii) *VTI1B*, (iii) *YEATS2* and (iv) *MFNG*. d) *FANCG*-targeting siRNA assays in BT-474 cells, which harbor *FANCG* amplifications, showing no significant changes in the mRNA expression levels of: (i) *KIRREL3*, (ii) *CKB*, (iii) *ALDH6A1* and (iv) *PCDHB6*.

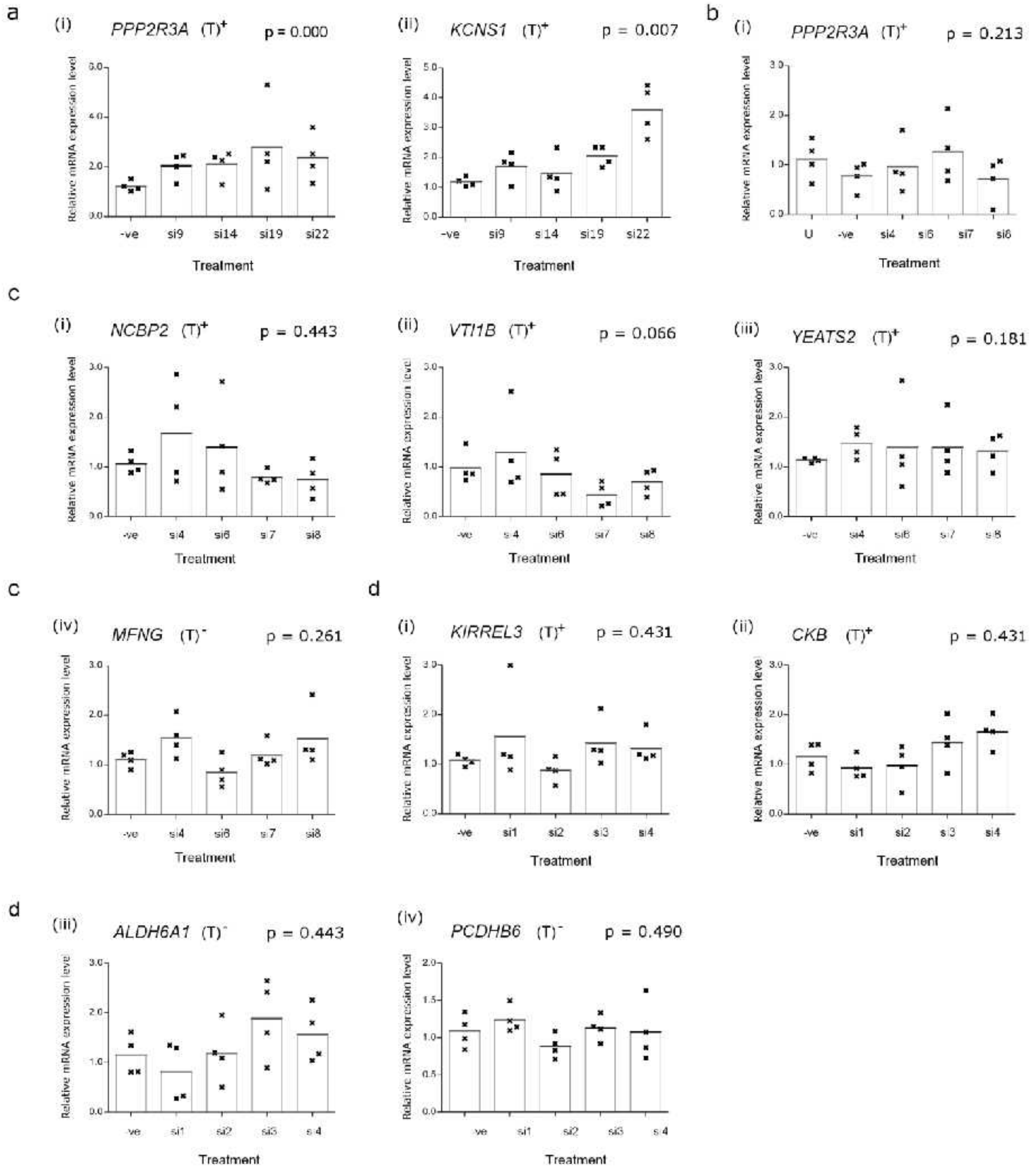

Data are results of four independent biological replicates and are represented as median and value ranges. (T) potential target genes, positively- (+) or negatively-regulated (-) by their respective regulator genes; (-ve) non-silencing negative siRNAs.

Supplementary Figure S6

The distribution of Pearson correlations of a random selection of acgh and expression profiles from the EAC dataset, Fisher Z-transformed (solid line), and overlain with the theoretical distribution for the correlations between two random variables (dashed line).

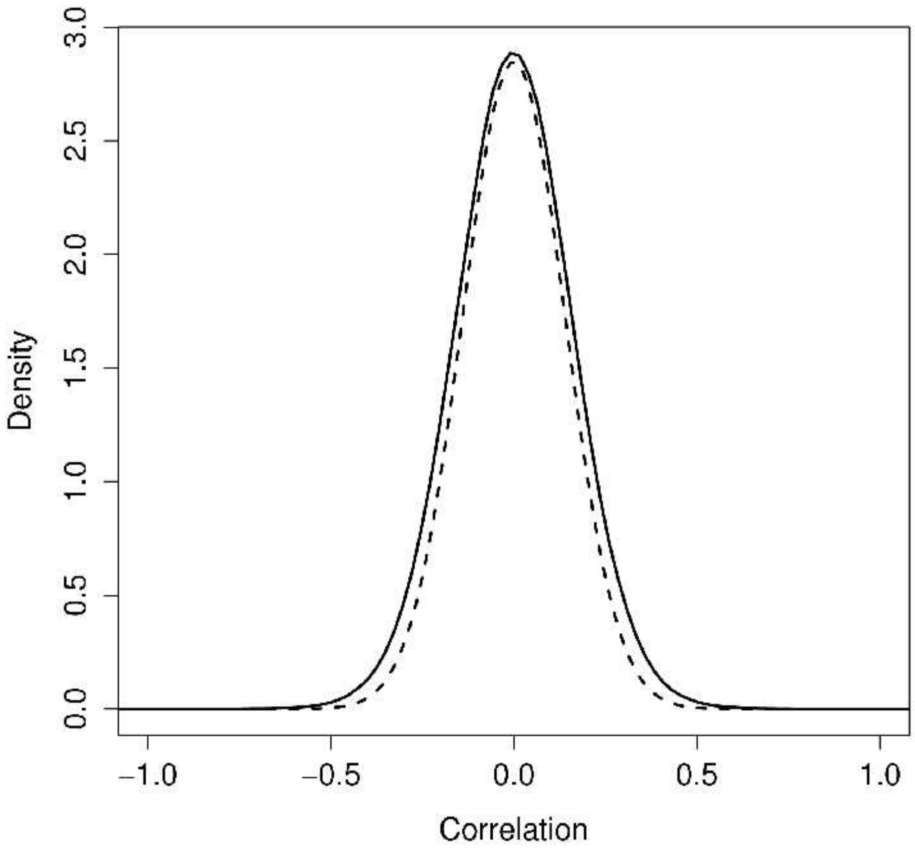

Supplement: File S1 — Supplementary Figures S1 to S6. (PDF) [file pone.0063780.s003.pdf]
