# Supplementary material for: Testing the Utility of an Integrated Analysis of Copy Number and Transcriptomics Datasets for Inferring Gene Regulatory Relationships
Source: PLoS One. 2013 May 30;8(5):e63780. doi: 10.1371/journal.pone.0063780 (PMC3667814; doi:10.1371/journal.pone.0063780)
Supplement: File S2 — Supplementary Tables S1 to S4. (PDF) [file pone.0063780.s004.pdf]

Supplementary Table S1

The 10 publicly available cancer datasets with matched genome-wide copy number and expression data used in the study. Dimensions are after probe mapping. * Data from Chitale *et al.*'s study were generated on two different expression array platforms, and data from each platform was treated separately as two datasets, so a total of 11 datasets were included in the multiple dataset analysis.

Authors	Number of samples	Number of probes	Cancer type	Accession numbers
Parris *et al.*	97	20738	Breast cancer: diploid breast carcinoma	GSE20486
Chin *et al.*	113	10943	Breast cancer	GSE8757
Crowder *et al.*	31	16202	Breast cancer: ER+ breast cancer	GSE15130<br>GSE15134
Sircoulomb *et al.*	27	15065	Breast cancer: *ERBB2*-amplified	GSE17907
Myllykangas *et al.*	46	8053	Gastric cancer: Intestinal, diffuse type	CG-EXP-26
Junnila *et al.*	10	16614	Gastric cancer: Intestinal, diffuse type	CG-EXP-49
Chitale *et al.*	174	9190; 9190 *	Lung adenocarcinoma	see link **
Medina *et al.*	8	6382	Lung cancer cell lines	GSE14079
Peters *et al.*	20	18036	Gastric adenocarcinoma	GSE19417
Goh *et al.*	54	18036	Esophageal adenocarcinoma	GSE20154

GSE: Accession numbers for data deposited on the Gene Expression Omnibus online database of the National Center for Biotechnology Information (NCBI); CG-EXP: Accession numbers for data deposited on Cancer GEnome Mine (CanGEM) online database of Genome Informatics Unit, Biomedicum Helsinki; **http://cbio.mskcc.org/Public/lung_array_data/.

Supplementary Table S2

Single dataset: List of top 20 predicted potential regulator genes; fdr, or the false discovery rate, is the Benjamini-Hochberg adjusted p-values.

Potential regulator genes	Chromosome	fdr
THEX1	8	0.0001
AKAP9	7	0.0015
PPP1R3B	8	0.0015
RPS4Y1	Y	0.0015
EGFR	7	0.0015
KIF3A	5	0.0015
CLDN23	8	0.0020
FDFT1	8	0.0030
MTMR9	8	0.0033
PEX1	7	0.0038
DKFZP564O0523	7	0.0049
GATAD1	7	0.0100
HSS00015817	8	0.0116
FLRT3	20	0.0203
*FGFR2**	10	0.0203
IL8RBP	2	0.0203
ABCC13	21	0.0219
ARMC3	10	0.0229
OSR2	8	0.0231
*ERBB2**	17	0.0231

* *FGFR2* and *ERBB2* were chosen for validation with their potential target genes.

Supplementary Table S3

Summary of all small interfering RNA sequences used for RNA interference assays, showing how their names were labeled in graphs.

siRNA target	Product name	Graph labels	Sequence
ARPC1A	Hs_ARPC1A_7	si7	TCCGCCATCCAGCATGACAAA
	Hs_ARPC1A_8	si8	CCCAATGCTCTTTAACTACGA
	Hs_ARPC1A_4	si4	CCTGGTGATCCTGAGAATTAA
	Hs_ARPC1A_6	si6	CAGTGGGTGAAAGCTCATGAA
ERBB2	Hs_ERBB2_9*	si9	AAGTGTGCACCGGCACAGACA
	Hs_ERBB2_14	si14	AACAAAGAAATCTTAGACGAA
	Hs_ERBB2_19*	si19	CGGCCCTAAGGGAGTGTCTAA
	Hs_ERBB2_22*	si22	TTCCAGATCCTGGGTACTGAA
FANCG	Hs_FANCG_3	si3	CCGGCTCGTTCGACAGGCCAA
	Hs_FANCG_4	si4	CTCATTGAGGTAGAATTACTA
	Hs_FANCG_1	si1	CCGCGGGAACTAACTCTTCAA
	Hs_FANCG_2	si2	CCCAGGTAATCGAGACACTTA
FGFR2	Hs_FGFR2_7	si7	CAGCATATGTGTAAAGATTTA
	Hs_FGFR2_15	si15	TTGCTTGGACTTAACTAGTTA
	Hs_FGFR2_16	si16	CCAGCTACTCCGGGAGGCTGA
	Hs_FGFR2_17	si17	ATCGCCGTTGTTCCTTCTGTA

* Rescue siRNAs

Supplementary Table S4

Conditions for RNA interference assays.

siRNA target	Cell line(s) used *	Cells seeded (cells/well)	[siRNA] (nM/well)	hiPerfect (µl/well)	Sources of cell lines
ARPC1A	AsPc1	$4x10^4$	25	3	American Type Culture Collection
ERBB2	BT474	$4x10^4$	15	3	DSMZ
	HSC39	$1x10^5$	15	6	Gift from K. Yanagihara †
	OE19	$4\text{-}8x10^4$	15	3	European Collection of Cell Cultures
FANCG	BT474	$4x10^4$	15	3	DSMZ
FGFR2	HSC39	$1x10^5$	25	6	Gift from K. Yanagihara †
	OE19	$4x10^4$	25	3	European Collection of Cell Cultures

* Tissue type: HSC39 (diffuse type gastric carcinoma); OE19 (esophageal adenocarcinoma); AsPc1 (pancreatic adenocarcinoma); BT474 (breast ductal carcinoma).

† Yanagihara, K. *et al.* Establishment and characterization of human signet ring cell gastric carcinoma cell lines with amplification of the c-myc oncogene. (1991) *Cancer Research*, 51, 381-386.
